# Aging impairs CD8 T cell responses in adoptive T-cell therapy against solid tumors

**DOI:** 10.3389/fimmu.2025.1484303

**Published:** 2025-01-24

**Authors:** Gulfiya Kadyrzhanova, Miho Tamai, Shukla Sarkar, Rajkumar Singh Kalra, Hiroki Ishikawa

**Affiliations:** Immune Signal Unit, Okinawa Institute of Science and Technology, Graduate University (OIST), Okinawa, Japan

**Keywords:** aging, adoptive T-cell therapy, cancer, CD8 T cells, Epas1

## Abstract

Age-associated defects in T cell-mediated immunity can increase the risk of cancers, but how aging influences adoptive T-cell therapy (ACT) for cancers remains unclear. Here, using a mouse model of melanoma, we demonstrate that aging diminishes anti-tumor activity of engineered CD8 T cells expressing a tumor-specific T cell receptor (CD8 TCR-T cells) in ACT for solid tumors. Aged CD8 TCR-T cells cannot control tumor growth in either young or aged mice. Aged CD8 TCR-T cells are unable to accumulate efficiently in tumors and have higher tendency to become terminally exhausted T cells with lower expression of endothelial PAS domain-containing protein 1 (Epas1) compared to young cells. Crispr-mediated ablation of *Epas1* promotes terminal exhaustion of young CD8 T cells in tumors, diminishing their anti-tumor activity in young mice. Conversely, retroviral expression of Epas1 enhances anti-tumor activity of aged CD8 TCR-T cells. These findings suggest that aging-induced reduction of Epas1 expression impairs anti-tumor activity of CD8 T cells in ACT against solid tumors, which can be therapeutically improved by expression of exogenous Epas1.

## Introduction

Age-related decline in T cell functions appears to increase risks of various infectious diseases and cancers in elderly people (1). Aging decreases naïve T cell abundance, while increasing numbers of dysfunctional T cell subsets, such as virtual memory T cells and senescent-like T cells (2). Additionally, aging diminishes diversity in the T cell receptor (TCR) repertoire in the circulating T cell population, leading to loss of specificity to specific pathogens and cancers (3–8). Furthermore, aging suppresses TCR signaling (9, 10) and metabolic reprogramming (11, 12) in T cell activation. Although there are conflicting reports and it remains controversial (12–14), several animal studies reported negative impacts of aging on anti-tumor CD8 T cell responses and immune checkpoint blockade responses (15–19).

Adoptive cell therapy (ACT) using engineered CD8 T cells is a promising approach to treat cancers. With this approach, autologous or allogenic CD8 T cells transduced with tumor antigen-specific TCRs (CD8 TCR-T cells) or chimeric antigen receptors (CAR-T cells) are cultured *in vitro* and then adoptively transferred into patients (20). CAR-T cells directly recognize cell surface antigens (21), whereas CD8 TCR-T cells recognize peptides presented by major histocompatibility complex (MHC) class I (22). Adoptive transfer of these engineered CD8 T cells provides a crucial solution to the lack of specificity for tumor antigens in the endogenous TCR repertoire of patients. Additionally, *in vitro* activation and clonal expansion of these engineered CD8 T cells can be optimized to yield a large number of cells for ACT. High efficacy of ACT with engineered CD8 T cells has been confirmed across numerous tumor models in both pre-clinical and clinical studies (23–28), but impact of aging on efficacy of such therapy is unknown.

In this study, we investigated effects of aging on anti-tumor efficacy of ACT with tumor-antigen-specific CD8 TCR-T cells in a mouse model of melanoma. We found that ACT with young CD8 TCR-T cells effectively reduces tumor growth in young mice, whereas ACT with aged CD8 TCR-T cells fails to control tumor growth in both young and aged mice. Furthermore, we show that Epas1 expression is significantly reduced in aged CD8 T cells, and loss of Epas1 impairs anti-tumor responses of young CD8 T cells in ACT. Moreover, retroviral expression of Epas1 improves anti-tumor responses of aged CD8 T cells in ACT. These results suggest that aging-induced downregulation of Epas1 negatively impacts ACT with engineered CD8 T cells.

## Materials and methods

### Mice

Young (8-16 weeks old) and aged (68-96 weeks old) C57BL/6 mice were obtained from Jackson Laboratory. OT-I mice (CD45.1^+^CD45.2^+^) were obtained by breeding of OT-I mice (CD45.1^−^CD45.2^+^) (Jackson, 003831) and B6SJL mice (CD45.1^+^CD45.2^−^) (Jackson, 002014). All mice were maintained under specific pathogen-free conditions. Female mice were used in all experiments. All animal experiments were approved by the Animal Care and Use Committee at Okinawa Institute of Science and Technology Graduate University.

### Isolation, activation, and culture of murine CD8 T cells

CD8 T cells were isolated from spleens of mice, by homogenizing them with a 70-μm cell strainer to release splenocytes, and the cell suspension was washed with MACS buffer. Erythrocytes were lysed by incubating the cell suspension in ammonium-chloride-potassium (ACK) buffer for 5 min. Then cells were resuspended in MACS buffer and centrifuged at 1,500 rpm for 5 min. CD8 T cells were negatively selected using a MojoSort Mouse CD8 T Cell Isolation Kit (Biolegend, 480035). CD8 T cells were then resuspended in complete RPMI-1640 media containing 2 mM L-glutamine, 1% HEPES (Life technologies; 15630080), 1 mM sodium pyruvate (Thermo Fisher, 11360070), 10 mM MEM non-essential amino acids (Thermo Fisher, 11140050), 0.05 mM 2-mercaptoethanol (Gibco, 21985-023), 10% fetal bovine serum (FBS) (Nichirei Bioscience, 173012) and 1% penicillin and streptomycin. CD8 T cells were activated for 24 h with an anti-CD3e antibody (5 μg/mL, Biolegend, 100340)-coated plate with soluble Ultra-LEAF purified anti-mouse CD28 antibody (2.5 μg/mL, Biolegend, 102116) and IL-2 (10 ng/mL, Biolegend, 575406). In some experiments, CD8 T cells were activated with Dynabeads Mouse T-activator CD3/CD28 beads (Gibco, 11456D).

### Retroviral vectors

pMIGR1-OT-I was generated by cloning a DNA cassette encoding TCRα-V2 and TCRβ-V5.2 linked to an intervening self-cleaving T2A peptide, obtained from TCR OT-I-2A.pMIGII plasmids (a gift from Dario Vignali (29) [Addgene, 52111]), into the pMIGR1 retroviral vector plasmid (Addgene, 27490). pMSCV-Epas1-Thy1.1 was generated by cloning Epas1 cDNA (Thermo Fisher, MMM1013-202767912) into pMSCV-IRES-Thy1.1 (Addgene, 17442).

### Retrovirus production

Platinum-E (Plat-E) cells were seeded in 100-mm culture dishes. Once they reach 50-70% confluency, they were transfected with 10 μg pMIGR1-OT-I or pMSCV-Epas1-Thy1.1 and 4.5 μg pCL-Eco retrovirus packaging vector (Addgene, 12371) using polyethylenimine (PEI: Polysciences, lnc, 24765). Briefly, plasmid DNA was mixed with PEI at a ratio of DNA: PEI=1:5 in 2.5 mL of Opti-MEM (Invitrogen, 31985-062), vortexed, and incubated for 30 min at room temperature (RT). This transfection mixture was added to walls of culture dishes without disturbing attached Plat-E cells. After 16-18 h, media were replaced with fresh media. GFP signals were checked after 48 h using fluorescent microscopy, to assess transfection efficiency. Virus supernatant was collected 72 h after transfection, and viral particles were concentrated by ultracentrifugation for 2-2.5 h at 24,000 g at 4°C with a Beckman Coulter Optima XPN-100 ultracentrifuge. The viral pellet was resuspended in complete RPMI media and used for retroviral transduction.

### Retroviral transduction

To generate OT-I TCR-T cells, CD8 T cells were transduced with MIGR1-OT-I retroviral vector. Retroviral transduction was performed as described previously (30). Non–treated cell-culture 24- or 48-well plates were coated with 0.5 mL of recombinant RetroNectin (20 μg/mL, Takara Bio) overnight at 37°C, washed in PBS, and blocked with 0.5 mL of 2% bovine serum albumin (BSA) in PBS. Retroviral suspension was added to the plate, which was spun at 2,000 g for 2 h at 32°C in pre-warmed Beckman Coulter Allegra X-12R centrifuge for RetroNectin-retrovirus binding. Then, the supernatant was aspirated. For retrovirus transduction, on day -1, CD8 T cells were activated with anti-CD3 antibody, anti-CD28 antibody and IL-2 for 24 h. On day 0, cells were resuspended in culture media supplemented with IL-2 (5 ng/mL), transferred to wells of a plate coated with RetroNectin-retrovirus (1 x 10^6^ cells/well in 24-well plates or 0.5 x 10^6^ cells/well in 48-well plates), and incubated until day 2. From day 3 to day 8, cells were cultured with media containing 10 ng/mL of both IL-7 (Biolegend, 577802) and IL-15 (Biolegend, 566301), but not IL-2, and media were refreshed every other day. OT-I TCR-T cells expanded until day 8 were used for adoptive transfer experiments. For transduction of CD8 T cells with MIGR1-OT-I and MSCV-Epas1-Thy1.1, cells activated with anti-CD3 antibody, anti-CD28 antibody and IL-2 for 2.5 days were first infected with MSCV-Epas1-Thy1.1. 1 day later, cells were subsequently infected with MIGR1-OT-I.

### CRISPR-based genome editing with nucleofection

To prepare a pair of two Epas1-specific gRNA, each crRNA targeting Epas1 (5’-GGCGACAATGACAGCTGACA-3’ or 5’-AGAAATCCCGTGATGCCGCG-3’) were mixed with TracRNA and incubated for 5 min at 95°C in a PCR thermocycler then cooled to RT. Negative control crRNA #1 (Integrated DNA Technologies) was used as a negative control. gRNA was mixed with Cas9 and incubated for 10 min at RT. CD8 T cells were isolated and incubated overnight in a complete RPMI-1640 medium supplemented with IL-7. Cells were collected, washed, and resuspended in P4 primary cell nucleofector solution. Naïve cell suspension was added to solution containing a pair of gRNA-Cas9 specific to Epas1 and loaded into the cuvette wells. Nucleofection was done using Lonza 4D Nucleofector Core Unit using DS189 program. Cells were then collected and recovered in CM supplemented with IL-7 in the incubator overnight.

### Analysis of cell numbers and viability

Cell numbers and viability were analyzed using a Muse Count & Viability Kit (Luminex, MCH100102) according to manufacturer instructions.

### Flow cytometry analysis

For analysis of cell surface molecules, cells were stained with fluorochrome-conjugated antibodies in PBS containing 2% FBS for 20 min on ice. For analysis of intracellular molecules, cells were subjected to antibody staining using a Foxp3 staining buffer set (eBioscience, 005253-00), according to manufacturer instructions. For analysis of intracellular cytokines, cells were cultured with phorbol 12-myristate 13-acetate (Sigma, P8139, 50 ng/mL), ionomycin (Sigma, I9657, 500 ng/mL), and brefeldin A (Biolegend, 420601, 5 μg/mL) for 6 h. Prior to antibody staining, cells were incubated with anti-Fc receptor-blocking antibody (anti-CD16/CD32; Biolegend, 101320) and NIR-Zombie (Biolegend, 423106). Anti-CD8a (53-6.7, Biolegend), anti-CD44 (IM7, Biolegend), anti-CD62L (MEL-14, Biolegend), anti-PD1 (RMP1-30, Biolegend), anti-Tim3 (RMT3-23, Biolegend), anti-CD45.1 (A20, Biolegend), anti-CD45.2 (104, Biolegend), anti-Tox (TXRX10, e-bioscience), anti-GzmB (GB11, Biolegend), anti-IFN-γ (XMG1.2, Biolegend), anti-Perforin (S16009A, Biolegend), anti-TNFα (MP6-XT22, Biolegend), and anti-Thy1.1 (OX-7, Biolegend) were used. Samples were analyzed using a BD FACS Aria III, and data analysis was performed using FlowJo software.

### Analysis of cytotoxicity and cell division of T cells co-cultured with tumor cells

B16-OVA cells in complete RPMI-1640 media were seeded into 96 well-plates (1 x 10^4^ cells in 100 μL media/well). Sixteen hours later, media were replaced with 200 μL of complete RPMI-1640 media containing T cells expressing OT-I TCR (3 x 10^4^ cells in 200 μL media/well). This makes a 1:3 target-to-effector ratio. Cells were cultured for 72 h in the absence of any cytokines. To evaluate cytotoxicity of T cells, after 72 h of co-culture, viability of B16-OVA target cells was assessed by staining cells with Annexin V (Biolegend, 640919) and ZombieNIR (Biolegend, 144 423106), followed by flow cytometry analysis. B16-OVA cells were distinguished from T cells by the absence of CD8 expression. In experiments evaluating division of T cells, prior to co-culture, T cells were stained with CytoTellRed (AAT Bioquest, 22255) or carboxyfluorescein succinimidyl ester (CFSE: Biolegend, 23801) for 20 min at 37°C. After co-culture, cells were collected, washed, and dye dilution was analyzed using flow cytometry.

### Tumor model

Mouse B16-OVA MO4 melanoma cell line (Sigma-Aldrich, SCC420) was cultured in the RPMI-1640 (Thermo Fisher, 11875093) growth medium supplemented with 10% FBS, MEM non-essential amino acids, 10 mM HEPES, 2-mercaptoethanol, 1mg/ml geneticin (Gibco, 10131035), penicillin and streptomycin (Sigma, P4333-100ML). A monolayer culture of B16-OVA cells was harvested with trypsin, washed twice with PBS, and resuspended in PBS. 2 x 10^5^ cells (in 100 μL PBS) were subcutaneously injected into the right flank of mice. Before injection, fur was removed from the injection site for measurement of tumor size. Mice were monitored, and tumor length (L: greatest longitudinal measurement) and width (W: greatest transverse measurement) were measured using digital caliper every other day. Tumor volume calculation: V=(L x W^2^)/2. During the analysis (until day 20 post-tumor injection), no mice died and did not have tumors beyond 20 mm in diameter, the humane endpoint for the analysis.

### Adoptive transfer of CD8 T cells

Adoptive transfer of OT-I TCR-T cells or OT-I T cells was performed by intravenously injecting cells into mice on day 8 after tumor transplantation. The tumor size on the day of adoptive transfer was below 2.5-5.0 mm in both length and width. OT-I TCR-T cells were prepared as described above and were subjected to flow cytometry analysis of GFP to assess retroviral transduction efficiency immediately before adoptive transfer. Subsequently, OT-I TCR-T cells including 10^6^ GFP^+^ cells were injected to each of C57BL/6 recipient mice. For adoptive transfer of Epas1-Crispr-treated OT-I T cells, OT-I T cells (CD45.1^+^CD45.2^+^) were nucleofected with Epas1-Crispr as described above, and then activated with anti-CD3 and anti-CD28 antibodies and IL-2 for 24 h. Cells were then cultured in the presence of IL-7 and IL-15 (10 ng/ml each) for 6 days. Subsequently, 10^6^ of these OT-I T cells were adoptively transferred into each of congenic recipient mice (CD45.1^−^CD45.2^+^).

### scRNA-seq

Lymphocytes isolated from tumors were stained with anti-CD8a antibody and ZombieNIR. Adoptively transferred cells (CD8^+^ZombieNIR^-^GFP^+^) were FACS-sorted. Single-cell suspensions were incubated with anti-mouse CD16/32 (TruStain fcX) antibody for 10 min at 4°C. TotalSeq-C0301 (Biolegend, 155861) and TotalSeq-C0302 (Biolegend, 155863) hashtag antibodies were used to stain aged and young cell suspensions, respectively, for 30 min at 4°C. Cell suspensions were washed three times, filtered through a cell strainer, and pooled. Samples were loaded into a 10X Genomics Chromium Next GEM. Library preparation was performed using Chromium next GEM single-cell 5’ reagent kits v2 (dual index) according to manufacturer instructions. Sequencing was performed on a NovaSeq 6000 SP (Illumina) with a target of 65,000 reads per sample.

### scRNA-seq data analysis

CellRanger single-cell software (10x Genomics, v6.1.2) was used for barcode processing, demultiplexing, and transcript counting to obtain a unique molecular identified (UMI) count matrix. Alignment was done on a mm10 reference genome. scRNA-seq data was analyzed using the R package, Seurat (by Satija Lab). Standard pre-processing was used to filter low-quality cells with feature counts fewer than 200 and more than 9500, excluding those with mitochondrial counts of over 5%. Data were normalized using the LogNormalize function. Next, FindVariableFeatures and ScaleData functions were employed before downstream analysis. Further principal component analysis (PCA) and RunTSNE with t-distributed stochastic neighborhood embedding (t-SNE) dimensional reduction were used, and cells were clustered. 831 young cells and 1,494 aged cells were detected for t-SNE visualization of Cd8a-expressing cell populations. FindMarkers was also used to identify differentially expressed genes in aged and young samples. Gene set enrichment analysis (GSEA) was performed based on genes ranked by fold changes in expression between aged and young OT-I TCR-T cells.

### Bulk RNA-seq

CD8 T cells treated with Epas1-Crispr or control-Crispr were FACS-sorted from tumor tissues. Total RNA was obtained from samples using an RNAdvance Cell V2 kit (Beckman Coulter). RNA concentration was measured on a Qubit Flex Fluorometer using an RNA HS assay kit (Thermo Fisher). A library was prepared using a QuantSeq3’ mRNA-Seq Library Prep Kit FWD for Illumina (Lexogen) with 4 ng of RNA per sample. Libraries were quantified using a Qubit 1x dsDNA HS assay kit on a Qubit Flex Fluorometer. Quality checking of libraries was performed using a High Sensitivity D5000 ScreenTape with a Tapestation 2200 (Agilent). Libraries were then pooled and subjected to sequencing on a NovaSeq 6000 (Illumina) with 1x100-bp reads.

### Immunoblot analysis

Proteins were extracted using RIPA buffer (Wako, 182-02451) with a protease inhibitor cocktail (Roche, 04693116001). Lysates were denatured with 5X loading buffer (250 mM Tris-HCl pH 6.8, 5% β-mercaptoethanol, 30% glycerol, 10% SDS, and 0.1% bromophenol blue) and resolved on SDS polyacrylamide gel electrophoresis. Proteins on the gel were transferred on PVDF (Trans-Blot Turbo™ Mini PVDF, Bio-Rad, 1704156) membrane. The PVDF membrane was washed with 1X PBS-T solution and immersed in sufficient volume of Ponceau S Staining Solution (Sigma Aldrich, P7170) for 10 minutes. Subsequently Ponceau Solution was decanted, and membrane was washed with distilled water until the background became clear. Membrane was taken on the white plate and images were captured. For antibody staining, membranes were blocked with 5% bovine albumin (Wako) in tris-buffered saline with 0.1% Tween-20. Anti-Epas1 antibody (NOVUS Bio, NB100-122SS) was incubated on membranes at 4°C overnight, followed by HRP-tagged secondary antibody incubation at room temperature for 2 h. Chemiluminescent signals were developed with Clarity ECL substrate (Bio-Rad, 170-5060) and detected using an iBright™ CL1500 Imaging System (Thermo).

### Bulk-RNA-seq data analysis

Raw fastqc files were checked for the quality of sequencing data using FastQC (v0.11.9), followed by alignment with bowtie2 to mouse reference genome GRCm38. Alignment files were processed using Samtools (v1.12). The number of reads that overlapped genes in Gencode reference was determined using featureCounts in Subread (v.2.0.1). The count output was read in RStudio. Differentially expressed genes were identified using the Walt test in the DEseq2 package between cells with Epas1-Crispr and control-Crispr.

### Statistical analysis

All statistical analysis were calculated using GraphPad Prism 10. P values <0.05 were considered as statistically significant. Repeated measures two-way ANOVA with Fisher’s LSD test, unpaired two-tailed t-tests and one-way ANOVA with Tukey’s multiple comparison test were used. Data are represented as means ± standard deviations (SD) or ± standard errors of the mean (SEM).

## Results

### Generation of tumor-specific CD8 TCR-T cells derived from young and aged mice

**Figure 1:**
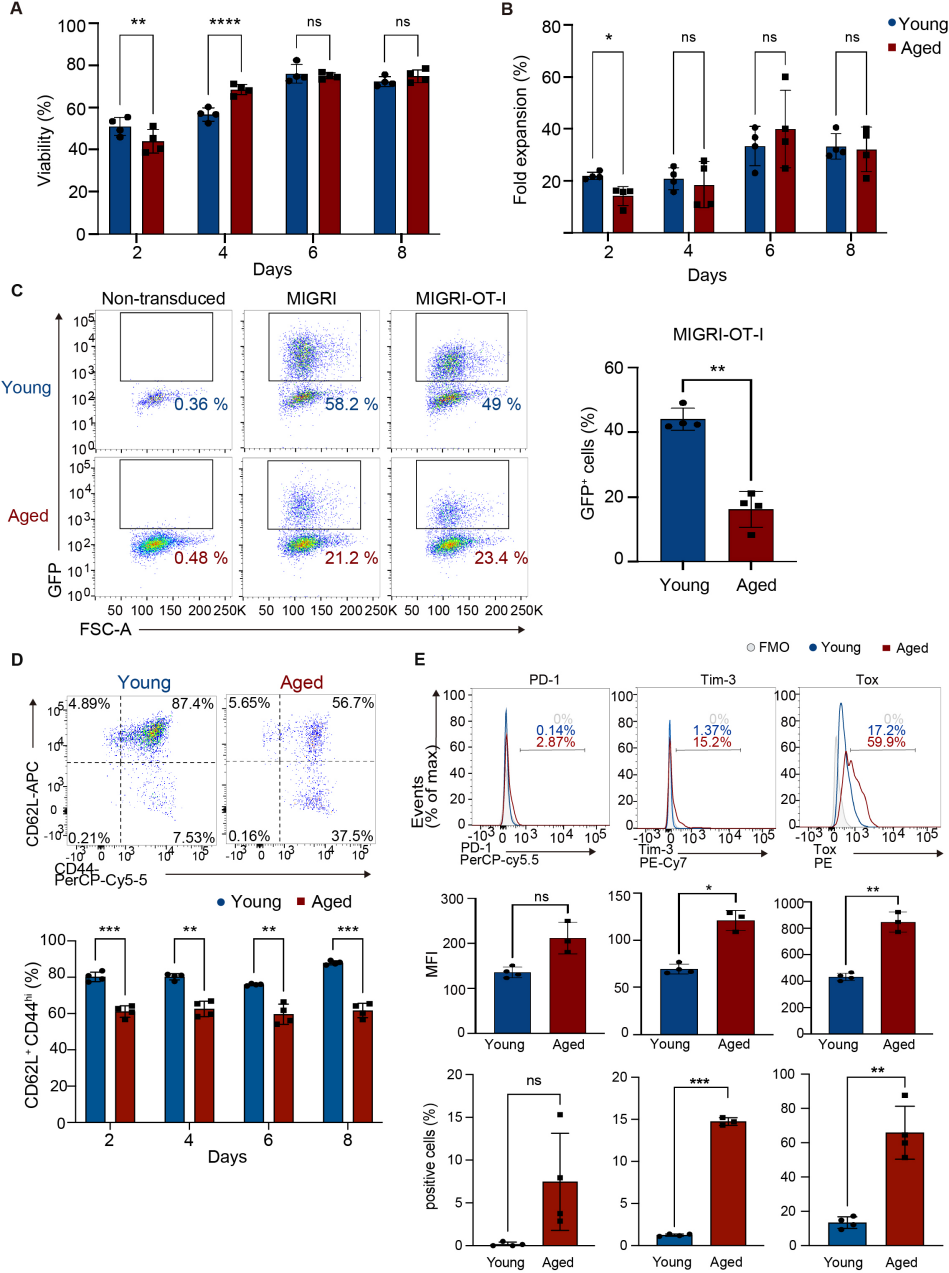
Generation of CD8 TCR-T cells from young and aged CD8 T cells. CD8 T cells isolated from young and aged mice were activated with anti-CD3, anti-CD28 antibodies, and IL-2 and transduced with a retroviral vector expressing OT-I TCR (MIGR1-OT-I) on day 0 and were expanded in media supplemented with IL-7 and IL-15. **(A, B)** Cell viability **(A)** and the fold increase in cell numbers **(B)** were assessed using a Muse cell analyzer. **(C-E)** Flow cytometry analysis of GFP expression on day 8 **(C)**, abundance of cells expressing CD44 and CD62L in GFP^+^ cells between day 2 and day 8 **(D)**, and expression of PD-1, Tim3, and Tox in GFP^+^ cells on day 8 **(E)**. The gating strategy for GFP^+^ cells is shown in **Supplementary Figure S1B**. Representative flow cytometry plots and histograms are shown. Graphs show percentages of cells expressing GFP **(C)**, CD62L^+^ CD44^hi^ cells **(D)**, and mean fluorescence intensity (MFI) and percentages of cells positive for each protein **(E)**. In C, CD8 T cells transduced with a control vector (MIGR1) and those that were non-transduced were also analyzed for comparison. FMO: fluorescence minus one. FMO for analysis of CD62L and CD44 expression is shown in **Supplementary Figure S1C**. **(A-C, E)** n = 4 per group, biological replicates. **(D)** n = 4 (young cells) and n = 3 (aged cells), biological replicates. **(A-E)** Error bars represent standard deviations (SD). Statistical analysis was performed using RM two-way ANOVA with an uncorrected Fisher’s LSD test **(A, B, D)** or unpaired t-test **(C, E)**. ns, not significant, *p <0.05, **p < 0.01, ***p<0.001, ****p<0.0001. Data are representative of 4 **(A-D)** or 3 **(E)** independent experiments.

We sought to investigate the effect of aging on ACT for solid tumors, using CD8 T cells transduced with an MHC class I-restricted TCR specific for chicken ovalbumin (OVA) antigen (OT-I TCR-T cells). To generate OT-I TCR-T cells, we isolated CD8 T cells from spleens of young (8-12 weeks) or aged (68-96 weeks) C57BL/6 mice and then activated these cells with TCR stimulation and infected them with a retroviral vector bi-cistronically expressing GFP reporter with OT-I TCRα and TCRβ chains linked with an intervening self-cleaving T2A peptide. Subsequently, we expanded these cells in the presence of cytokines IL-7 and IL-15 according to a protocol optimized for the expansion of CAR-T cells in a previous study (30). Aged T cells exhibited steady growth with high cell viability, similar to young T cells (**Figures 1A, B**, **Supplementary Figure S1A**). Flow cytometry analysis showed that transduction efficiency of OT-I TCR was approximately 20% in aged T cells and 40% in young T cells, confirmed by GFP reporter expression (**Figure 1C**).

CAR-T cells with a central memory cell phenotype characterized by expression of CD62L and CD44 (CD62L^+^ CD44^hi^) exhibit high anti-tumor activity (31). Flow cytometry analysis showed that aged OT-I TCR-T cells had lower frequency of cells with the CD62L^+^ CD44^hi^ phenotype and higher frequency of cells with the CD62L^-^ CD44^hi^ effector phenotype than young OT-I TCR-T cells (**Figure 1D**, **Supplementary Figures S1B–D**). Analysis of exhaustion-related molecules revealed no induction of PD-1 and only minimum induction of Tim3 and Tox in aged TCR-T cells (**Figure 1E**).

**Figure 2:**
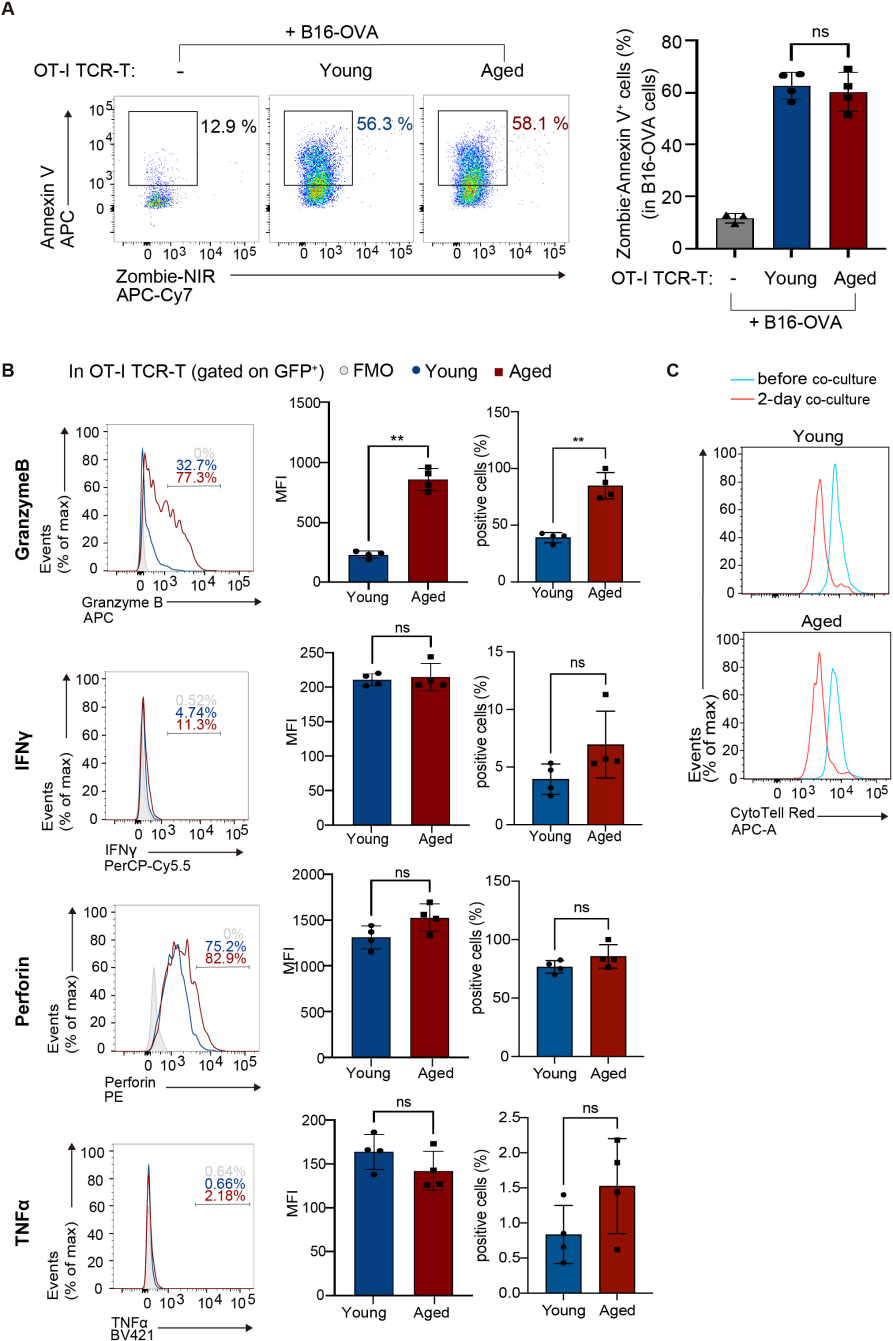
Aged CD8 TCR-T cells have normal cytolytic functions *in vitro*. OT-I TCR-T cells were generated by transducing MIGR1-OT-I into young or aged CD8 T cells as in **Figure 1**. Following an 8-day culture, transduction efficiency (percentages of GFP-expressing cells) was assessed using flow cytometry. Subsequently, OT-I TCR-T cells were co-cultured with B16-OVA target cells at a ratio of GFP^+^ T cells to targets of 1 to 3. **(A)** Flow cytometry analysis of Annexin V and Zombie NIR staining in B16-OVA cells (gated on CD8^-^: the gating strategy is shown in **Supplementary Figure S2A**) co-cultured with OT-I TCR-T cells for 3 days. B16-OVA cells cultured alone for 3 days were also analyzed for comparison. **(B)** Flow cytometry analysis of expression of granzyme B, perforin, IFN-γ, and TNF-α in OT-I TCR-T cells (gated on CD8^+^ GFP^+^: the gating strategy is shown in **Supplementary Figure S2B**) co-cultured with B16-OVA for 1 day. Graphs show MFI and percentages of cells positive for each protein. **(A, B)** n = 4 per group, biological replicates. Error bars represent the SD. Statistical analysis was performed using one-way ANOVA with Tukey’s multiple comparisons test **(A)** or unpaired t-tests **(B)**. ns, not significant, **p < 0.01. **(C)** Flow cytometry analysis of CTV dilution. OT-I TCR-T cells were stained with CTV, and CTV dilution was analyzed before co-culture and after 2 days of co-culture. **(A-C)** Data are representative of 3 **(A)** or 2 **(B, C)** independent experiments.

Next, to evaluate their anti-tumor responses *in vitro*, young and aged OT-I TCR-T cells expanded were co-cultured with B16 melanoma-expressing OVA antigen (B16-OVA cells). Analysis of dead cells stained with Zombie dye and Annexin V revealed that aged OT-I TCR-T cells killed target B16-OVA cells as efficiently as young OT-I TCR-T cells (**Figure 2A**). Perforin, IFN-γ, and TNF-α were expressed at similar levels in young and aged OT-I TCR-T cells co-cultured with B16-OVA cells, while granzyme B expression was significantly higher in aged OT-I TCR-T cells (**Figure 2B**). Moreover, young and aged OT-I TCR-T cells showed comparable levels of proliferation upon co-culture with B16-OVA cells, as shown in a dye dilution assay (**Figure 2C**). These results indicate that TCR-T cells engineered from aged CD8 T cells are expandable, do not show prominent exhaustion phenotypes, and are able to exert cytotoxic functions on target tumor cells *in vitro* as young cells. However, they exhibit decreased ability to develop a central memory phenotype.

### Aging diminishes anti-tumor activity of CD8 TCR-T cells *in vivo*

**Figure 3:**
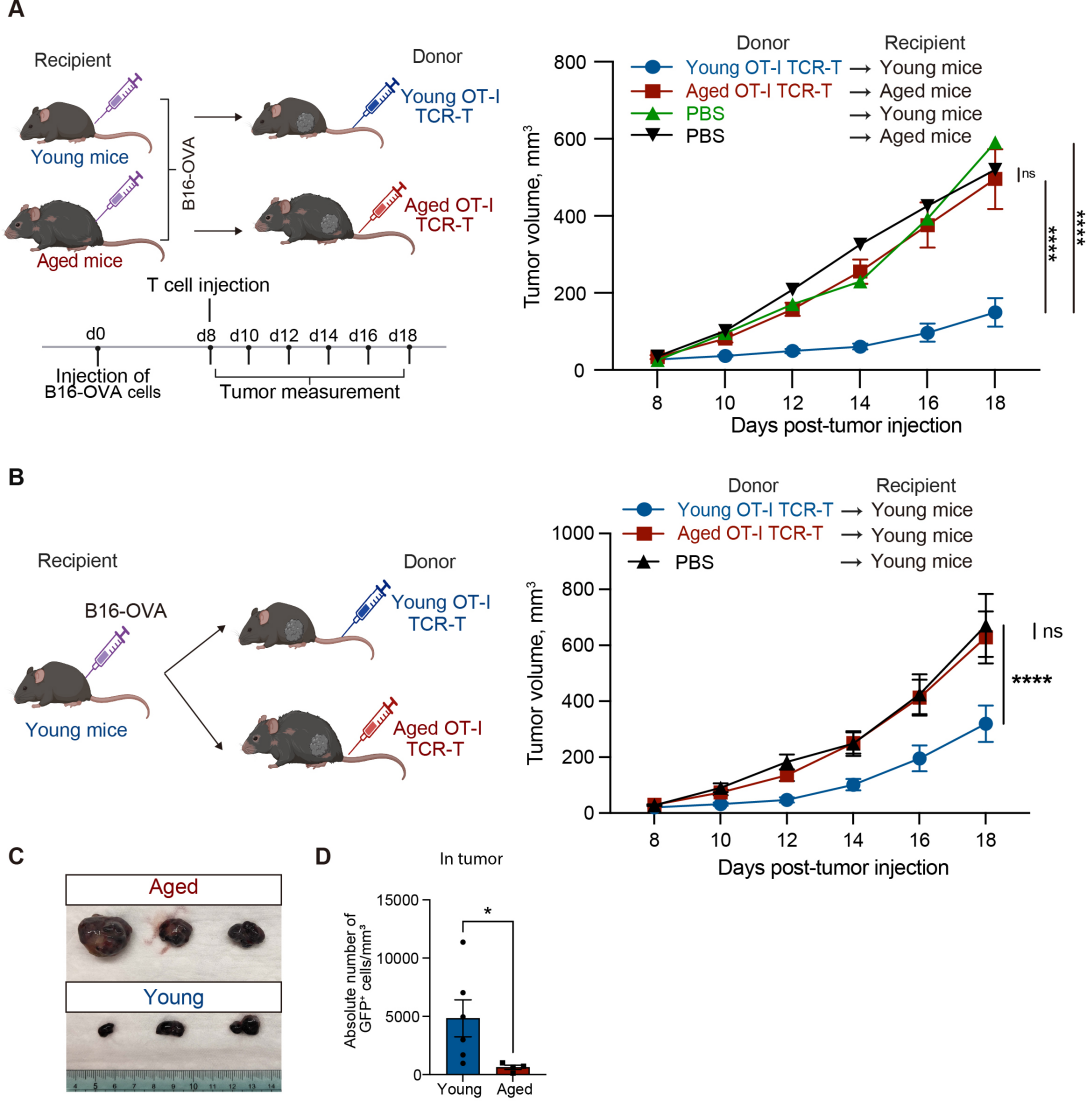
Aged CD8 TCR-T cells fail to control tumor growth in both young and aged mice. **(A, B)** Young and aged C57BL/6 mice were subcutaneously injected with 2 x 10^5^ B16-OVA cells on day 0. Eight days later, young and aged OT-I TCR-T cells were adoptively transferred into young and aged tumor-bearing mice, respectively **(A)** or into young tumor-bearing mice **(B)**. Adoptive transfer was performed by intravenously injecting cells including 10^6^ GFP^+^ cells after GFP expression was assessed by flow cytometry. Mice injected with PBS instead of adoptive cell transfer were also analyzed for comparison. Left schematic diagrams show the experimental design (created with BioRender.com). Right graphs show results of tumor volume measurements. **(A)** n = 10 (aged tumor-bearing mice), n = 8 (young tumor-bearing mice), and n = 3 (PBS). **(B)** n = 13 (mice transferred with aged cells), n = 17 (mice transferred with young cells), n = 12 (PBS). Results are shown as mean tumor volume ± SEM. Statistical analysis was performed using two-way RM ANOVA with uncorrected Fisher’s LSD test. ns, not significant, ****p<0.0001. Data are pooled from 2 **(A)** or 4 **(B)** independent experiments. **(C)** Photos of tumors isolated from mice analyzed as in **(B)** on day 20. n = 3 per condition. **(D)** Absolute numbers of adoptively transferred CD8^+^GFP^+^ T cells in tumors were analyzed by flow cytometry. n = 6 (young cells), n = 4 (aged cells). Statistical analysis was performed using unpaired t-tests. *p < 0.05. Data are representative of 2 independent experiments.

Next, we evaluated the effect of aging on anti-tumor activity of OT-I TCR-T cells *in vivo*. We subcutaneously injected B16-OVA tumor cells into young or aged C57BL/6 mice. On day 8, when tumor volume reached about 50 mm^3^, young and aged OT-I TCR-T cells were adoptively transferred into young and aged mice, respectively (**Figure 3A**). This revealed that adoptive transfer of young OT-I TCR-T cells significantly inhibited tumor growth in young mice, whereas aged OT-I TCR-T cells did not affect tumor growth in aged mice (**Figure 3A**), suggesting that aging reduces anti-tumor activity of CD8 TCR-T cells *in vivo*.

The diminished anti-tumor efficacy of adoptive transfer of CD8 TCR-T cells in aged mice might be attributed to age-related intrinsic changes in T cells. To address this possibility, we next compared anti-tumor activity of young and aged OT-I TCR-T cells in the same *in vivo* setting, using young tumor-bearing mice as recipients for adoptive transfer (**Figure 3B**). We chose this experimental setting because it has been suggested that certain phenotypes in aged T cells persist in a young tissue microenvironment, whereas young T cells acquire age-related phenotypes in an aged tissue microenvironment (32). We found that adoptive transfer of aged OT-I TCR-T cells did not inhibit tumor growth in young mice, unlike the effect observed with young OT-I TCR-T cells (**Figures 3B, C**). Moreover, the frequency of aged OT-I TCR-T cells in tumor tissues was significantly lower than that of young cells (**Figure 3D**). These results suggest that aging of CD8 T cells alone is sufficient to reduce their ability to control tumors in CD8 TCR-T therapy.

### Age-related changes in CD8 TCR-T cell anti-tumor responses

**Figure 4:**
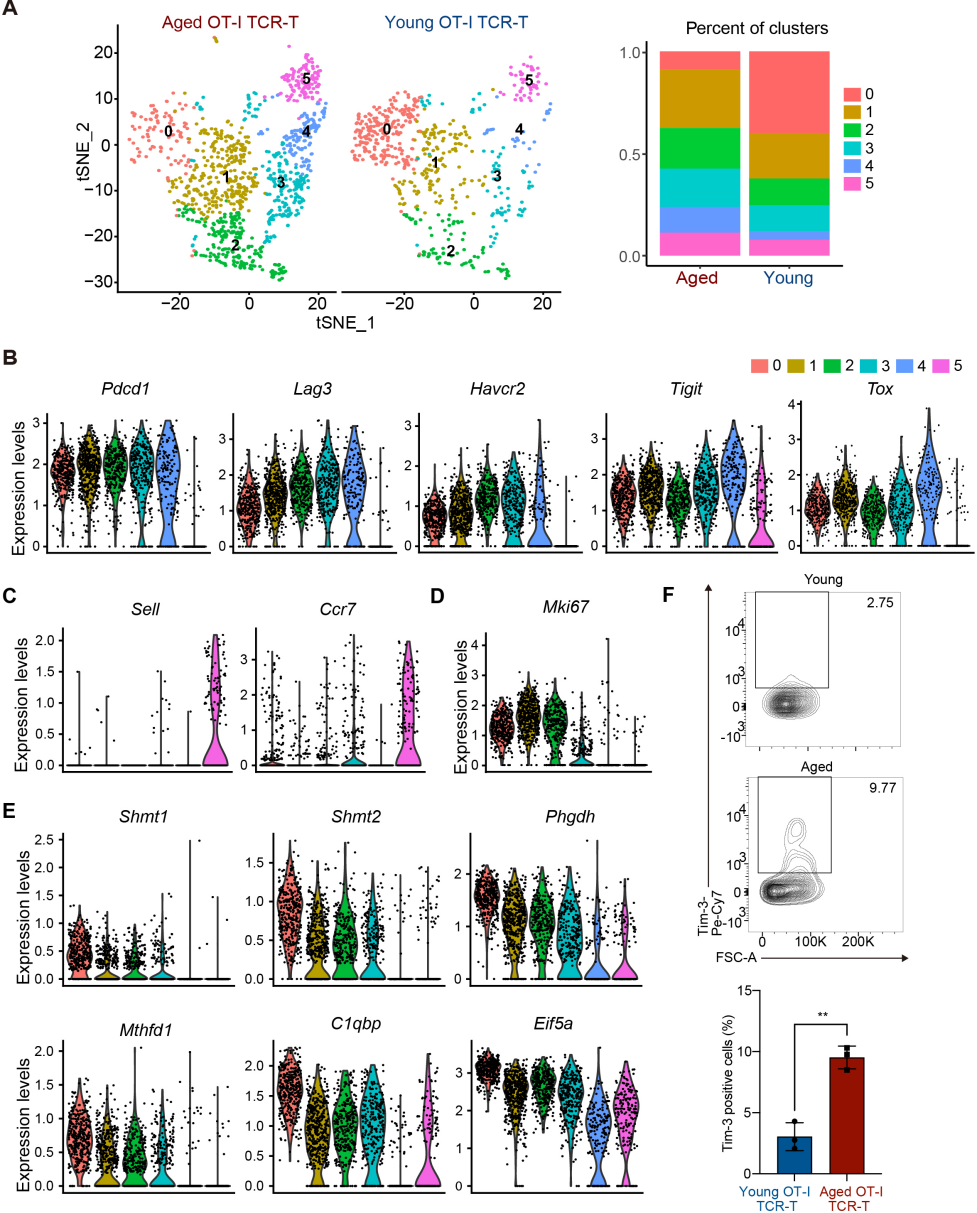
Aged CD8 TCR-T cells are prone to become Tex-like cells in tumors. **(A-F)** On day 0, young C57BL/6 mice were each subcutaneously injected with 2 x 10^5^ B16-OVA cells. On day 8, mice were each subjected to adaptive transfer with young or aged OT-I TCR-T cells, including 10^6^ GFP^+^ cells. On day 20, CD8^+^GFP^+^ OT-I TCR-T cells were FACS-sorted from tumors (n = 4, young cell transfer; n = 3, aged cell transfer), pooled in each group, and were subjected to scRNA-seq analysis. **(A)** t-SNE visualization showing young and aged OT-I TCR-T cells were grouped into clusters 0 to 5. Cells expressing *Cd8a* were used for analysis. The left panel shows t-SNE plots. The right panel presents a stacked bar graph showing percentages of cells in each t-SNE cluster. **(B-E)** Violin plots show expression of exhaustion-related genes (*Tox*, *Pdcd1*, *Lag3*, *Havcr2*, and *Tigit*) **(B)**, central memory-related genes (*Sell* and *Ccr7*) **(C)**, a proliferation marker gene, *Mki67* **(D)**, and genes highly expressed specifically in cluster 0 (*Shmt1*, *Shmt2*, *Phgdh*, *Mthfd1*, *C1qbp*, and *Eif5a*) **(E)**. **(F)** On day 20, Tim3 expression inf CD8^+^GFP^+^ OT-I TCR-T cells (gating strategy is shown in **Supplementary Figure S3B**) in tumors was analyzed using flow cytometry. n = 5 (mice transferred with young cells) and n = 3 (mice transferred with aged cells). The graph shows percentages of cells positive for Tim3 expression. Error bars represent SD. Statistical analysis was performed using unpaired t-tests. **p <0.01. Data are representative of 2 independent experiments.

To understand the mechanism underlying the cell-intrinsic defect in anti-tumor response of aged CD8 TCR-T cells, we next compared gene expression profiles of young and aged OT-I TCR-T cells in tumor tissues using scRNA-seq analysis. On day 12 after the adoptive transfer of young or aged OT-I TCR-T cells into young recipient mice with B16-OVA tumors, we collected tumor-infiltrating lymphocytes and sorted OT-I TCR-T cells (CD3^+^, CD8^+^, ZombieNIR^-^ and GFP^+^). We performed scRNA-seq analysis using pooled OT-I-TCR-T cells collected from these mice. t-distributed stochastic neighborhood embedding (t-SNE) analysis of cells expressing *Cd8* revealed that aged and young OT-I TCR-T cells were categorized into six clusters (clusters 0-5) (**Figure 4A**). Of note, aged OT-I TCR-T cells exhibited higher frequencies in clusters 1-5 and a lower frequency in cluster 0, compared to young OT-I TCR-T cells (**Figure 4A**).

PD-1-expressing exhausted T cells in tumor microenvironment exhibit distinct phenotypes, including Tim3^-^ progenitor exhausted (Tpex) and Tim3^+^ terminally exhausted T (Tex) phenotypes (33). In differentiation of Tpex cells into Tex cells, cells lose cell proliferation capacity and increase expression of multiple inhibitory receptors, such as Tim3, Lag3, and Tigit (34). In our scRNA-seq data, exhaustion-related genes *Pdcd1* and *Tox* were highly expressed in cells among clusters 0 to 4, but not in cluster 5 (**Figure 4B**). Cells in cluster 5 expressed homing receptor genes (*Sell* and *Ccr7*) (**Figure 4C**) but exhibited little to no expression of effector molecule genes (*Gzmb*, *Ifng*, and *Prf1*) (**Supplementary Figure S3A**), suggesting that they do not directly participate in anti-tumor responses. Cells in cluster 0 expressed lower levels of *Havcr2* (encoding Tim3), *Lag3*, and *Tigit* than many cells in clusters 1-4 (**Figure 4B**), while expressing the cell proliferation marker gene *Mki67* (**Figure 4D**), suggesting that they are Tpex-like cells. Along with high expression of multiple inhibitory receptors, cells in clusters 1 and 2 expressed Mki67, whereas cells in clusters 3 and 4 did not (**Figure 4D**), suggesting that cells in cluster 1 and 2 are cells differentiating to Tex, while cells in clusters 3 and 4 are Tex-like cells.

We also found that cells in cluster 0 exhibited increased expression of genes related to one-carbon metabolism, such as *Shmt1*, *Shmt2*, *Phgdh*, *Psat1*, and *Mthfd1* (35), and genes related to mitochondrial oxidative phosphorylation and cell cycle regulation, such as *C1qbp* and *Eif5a, respectively* (36, 37) (**Figure 4E**). Flow cytometry analysis confirmed that Tim3 expression levels in aged TCR-T cells in tumors were higher than in young TCR-T cells (**Figure 4F**, **Supplementary Figure S3B**). Taken together, these results suggest that aged CD8 TCR-T cells may lose the capacity to maintain Tpex population and are more prone to differentiate to Tex cells compared to young CD8 TCR-T cells in anti-tumor responses.

### Age-related changes in gene expression in anti-tumor CD8 TCR-T cell responses

**Figure 5:**
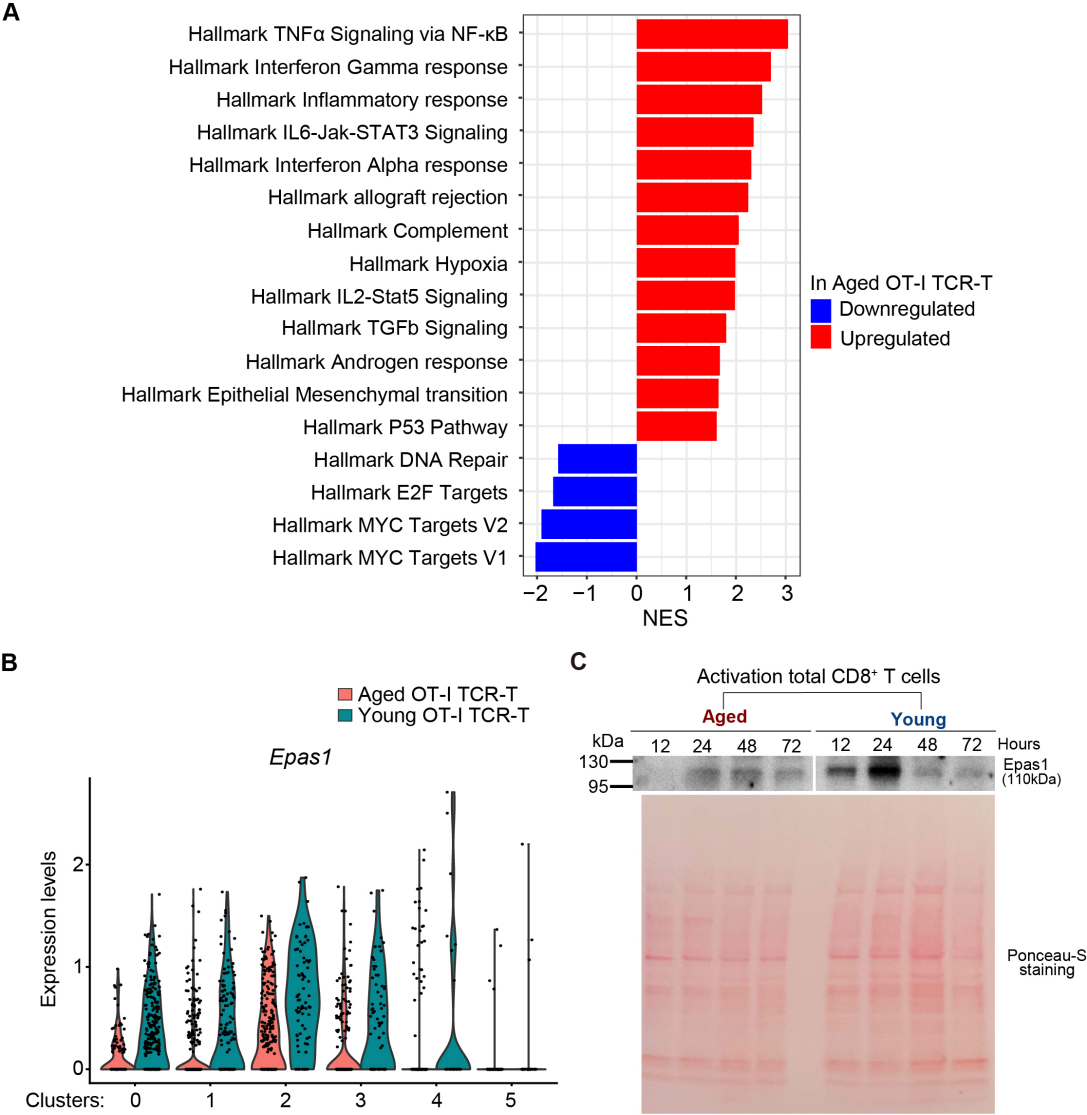
Epas1 expression is reduced in intra-tumoral aged CD8 TCR-T cells. **(A)** GSEA was performed using scRNA-seq data obtained in **Figure 4**. Red and blue bars represent upregulated and downregulated gene sets in aged OT-I TCR-T cells compared to young OT-I TCR-T cells. **(B)** Analysis of DEGs (adjusted p values <0.05) in scRNA-seq data obtained in **Figure 4** revealed significantly lower Epas1 expression in aged cells in clusters 0 to 4. The violin plot shows Epas1 expression in young and aged OT-I TCR-T cells. **(C)** CD8 T cells isolated from spleens of young and aged C57BL/6 mice were activated with anti-CD3 and anti-CD28 antibodies and IL-2. Cells were subjected to immunoblot analysis using an antibody against Epas1 at indicated time points. As a loading control, the blot was stained with Ponceau-S. Data are representative of 2 independent experiments.

We next performed gene set enrichment analysis (GSEA) of genes ranked by fold changes in expression between aged and young OT-I TCR-T cells in our scRNA-seq data. This showed that aged OT-I TCR-T cells exhibit upregulation of gene sets characteristic of TNF-α signaling, interferon gamma response, inflammatory response, and IL6-JAK-STAT3 signaling (**Figure 5A**). Enhanced responses to inflammatory cytokines in aged TCR-T cells may reflect enhanced production of inflammatory cytokines by other cell types in response to uncontrolled tumor growth. GSEA also revealed significant downregulation in hallmark gene sets of Myc targets in aged OT-I TCR-T cells compared to young cells (**Figure 5A**). Consistent with this, we identified *endothelial PAS domain protein 1* (*Epas1*, also known as *hypoxia inducing factor 2a* [*Hif2a*]), which promotes c-Myc functions by inducing c-Myc-Max complex (38), as the transcription factor gene most significantly downregulated in aged T cells in clusters 0-4 (**Figure 5B**). Furthermore, immunoblot analysis showed that TCR stimulation-induced Epas1 protein expression in aged CD8 T cells activated *in vitro* was lower than in young CD8 T cells (**Figure 5C**). These results suggest that an impaired Epas1-dependent pathway is a major age-related defect in tumor-specific CD8 T responses.

### Epas1 facilitates anti-tumor CD8 T cell responses

**Figure 6:**
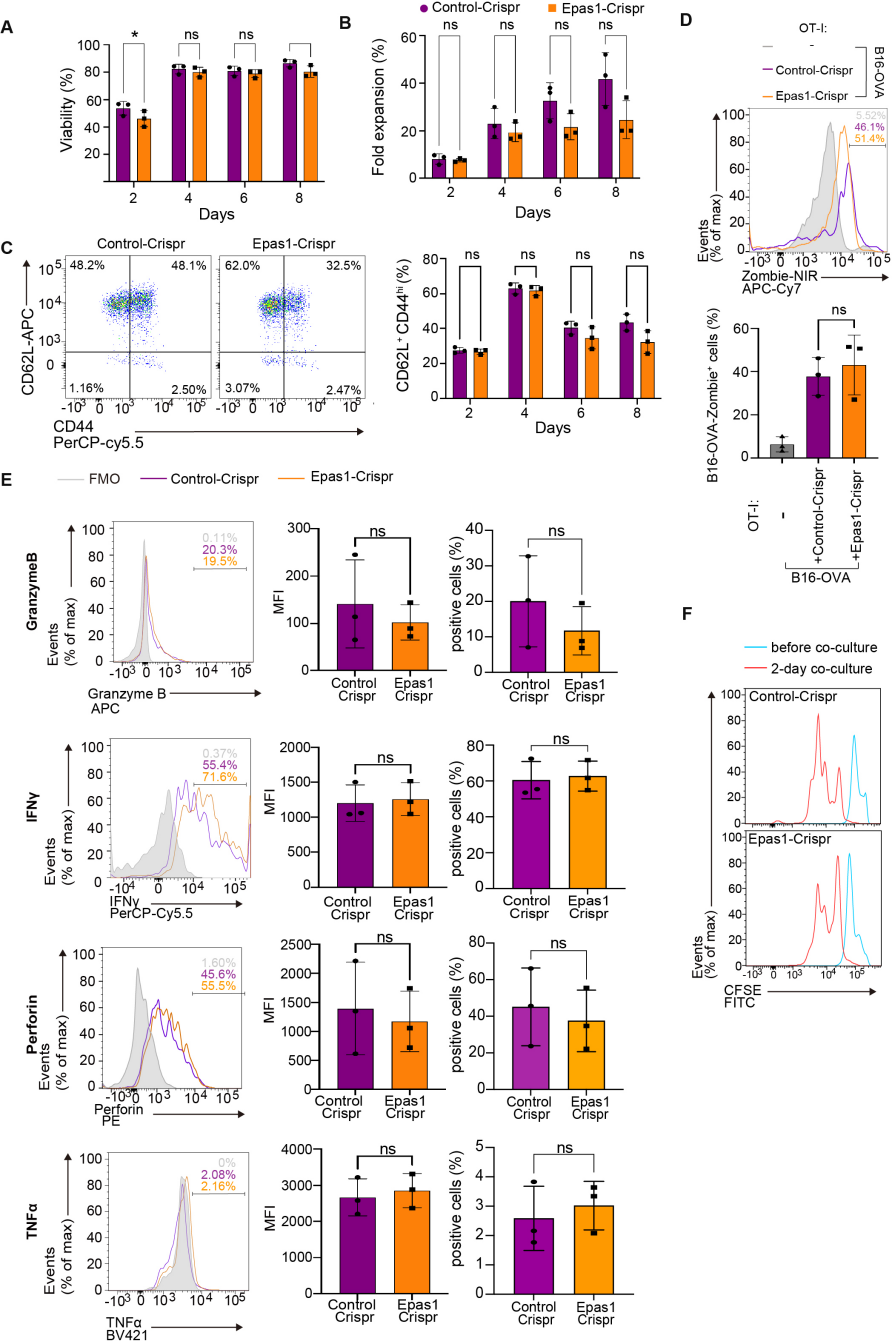
Epas1 is dispensable for activation and cytolytic functions of CD8 T cells *in vitro.* CD8 T cells were isolated from spleens of young OT-I mice (OT-I T cells) and were nucleofected with Crispr ribonucleoproteins targeting *Epas1* (Epas1-Crispr) or control ribonucleoproteins (control-Crispr) on day 0, before being cultured overnight. On day 1, cells were activated with anti-CD3 and anti-CD28 antibodies and IL-2 for 24 h. From day 2 to day 8, cells were cultured without TCR stimulation in the presence of IL-7 and IL-15. **(A, B)** Cell viability **(A)** and fold increase in cell numbers **(B)** were assessed using a Muse cell analyzer every other day between day 2 and day 8. **(C)** Flow cytometry analysis of expression of CD62L and CD44 in OT-I T cells (gating strategy is shown in **Supplementary Figure S4C**) was conducted every other day between day 2 and day 8. Graph shows the percentage of cells with a CD62L^+^CD44^hi^ (central memory: CM) phenotype. **(D-F)** On day 8, OT-I T cells were co-cultured with B16-OVA cells at a ratio of 1 to 3. **(D)** Flow cytometry analysis of Zombie NIR staining in B16-OVA cells (gated on CD8−: gating strategy is shown in **Supplementary Figure S4F**) co-cultured with OT-I T cells for 3 days. B16-OVA cells cultured alone for 3 days were also analyzed for comparison. **(E)** Flow cytometry analysis of expression of granzyme B, perforin, IFN-γ, and TNF-α in OT-I T cells (gated on CD8+: gating strategy is shown in **Supplementary Figure S4G**) co-cultured with B16-OVA for 24 h. **(F)** Flow cytometry analysis of CFSE dilution. OT-I T cells were stained with CFSE, and CFSE dilution was analyzed before and after 2 and 3 days of co-culture. Graphs show mean fluorescence intensity (MFI) and percentages of cells positive for each protein. **(A-F)** n = 3 per group. Error bars represent SD. Statistical analysis was performed with RM two-way ANOVA with Fisher’s LSD test **(A-C)** or unpaired t-tests **(E)**. ns, not significant, *p <0.05. Data are representative of 2 independent experiments.

We next sought to explore the possibility that reduction of Epas1 expression is involved in impaired anti-tumor responses of aged CD8 TCR-T cells. A previous study reported that overexpression of Epas1 promotes anti-tumor CD8 T cell responses (39), but the role of endogenous Epas1 in CD8 T cells remains unknown. To address this, we first investigated the effect of Epas1 deficiency on CD8 T cell activation and cytotoxicity on target tumor cells *in vitro*. We electroporated total CD8 T cells isolated from young OT-I transgenic mice (OT-I T cells) with Crispr/Cas9 protein and guide RNA (gRNA) targeting *Epas1* (Epas1-Crispr). Treatment with Epas1-Crispr significantly reduced expression of *Epas1* mRNA (**Supplementary Figure S4A**). Subsequently, we activated Epas1-Crispr-treated cells with TCR stimulation and expanded them in growth media supplemented with IL-7 and IL-15. We observed comparable growth and viability between cells treated with Epas1-Crispr or control-Crispr (Crispr/Cas9 protein and negative control gRNA) (**Figures 6A, B**, **Supplementary Figure S4B**). Epas1-Crispr treatment also did not affect the abundance of CD62L^+^ CD44^hi^ central memory cells (**Figure 6C**, **Supplementary Figures S4C–E**). Moreover, upon co-culturing with B16-OVA cells, OT-I T cells with Epas1-Crispr treatment exhibited normal, cytotoxicity on target cells (**Figure 6D**, **Supplementary Figure S4F**), expression of perforin, GzmB, IFN-γ, and TFN-α (**Figure 6E**, **Supplementary Figure S4G**), and proliferation (**Figure 6F**). These results indicate that Epas1 is not essential for CD8 T cell activation, proliferation, and anti-tumor cytotoxic activity *in vitro*.

**Figure 7:**
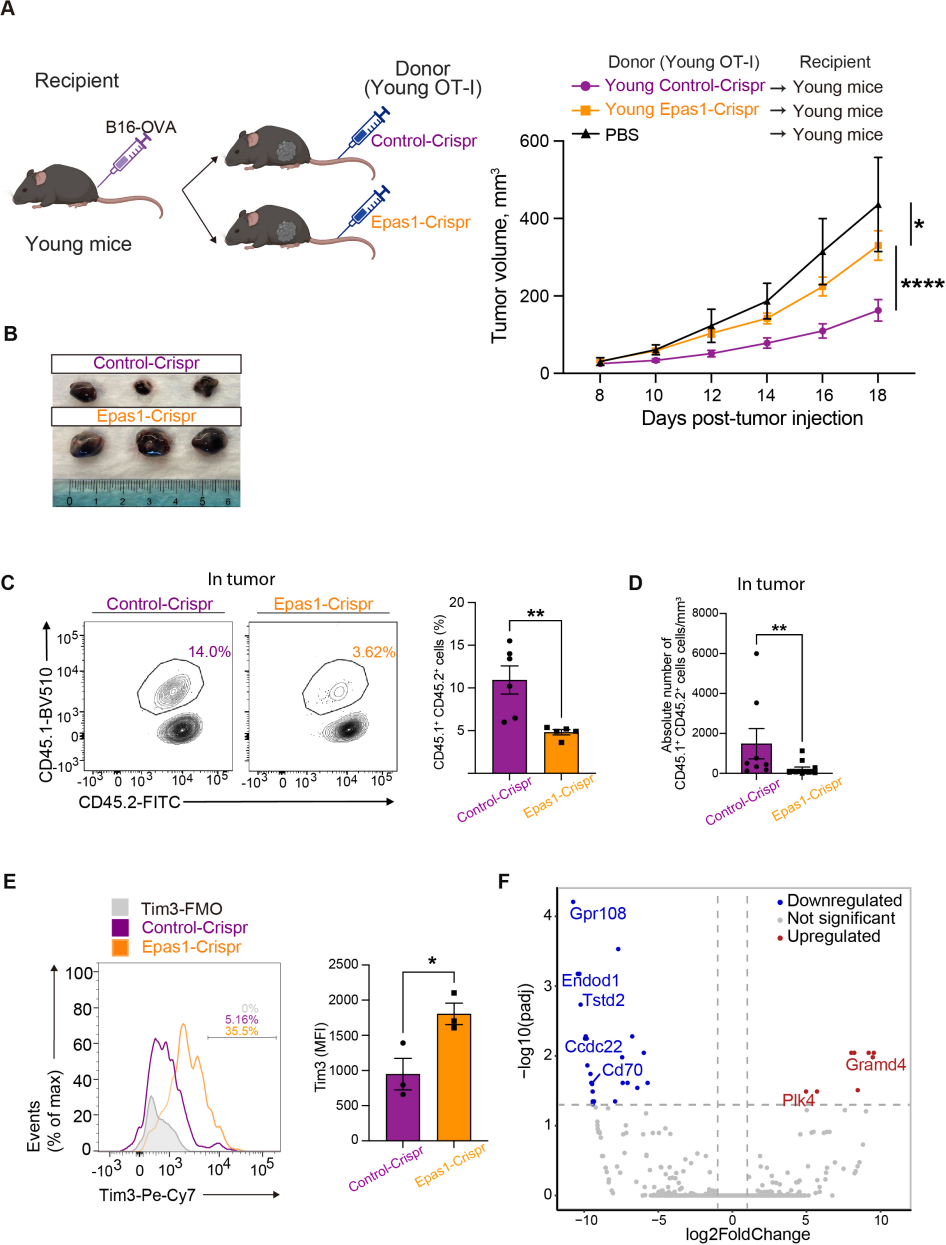
Epas1 is essential for the anti-tumor CD8 T cell response. Young OT-I T cells (CD45.1^+^CD45.2^+^) were nucleofected with Crispr ribonucleoproteins targeting *Epas1* (Epas1-Crispr) or control ribonucleoproteins (control-Crispr), and then activated with anti-CD3 and anti-CD28 antibodies and IL-2 for 24 h. Cells were cultured in the presence of IL-7 and IL-15 for 6 days. Subsequently, 10^6^ of these OT-I T cells were adoptively transferred into young congenic recipient mice (CD45.1−CD45.2^+^) that had been subcutaneously injected with 2 x 10^5^ of B16-OVA cells 8 days earlier (day 0). Mice injected with PBS instead of adoptive cell transfer were also analyzed for comparison. **(A)** Left, schematic diagrams show the experimental design (created with BioRender.com). Right, graphs show results of tumor volume measurements. Mice that received OT-I T cells with Epas1-Crispr (n = 23) or control-Crispr (n = 22) were analyzed. Data are pooled from four independent experiments. Results are shown as mean tumor volume ± SEM. Statistical analysis was performed using RM two-way ANOVA with uncorrected Fisher’s LSD test. *p <0.05, ****p < 0.0001. **(B)** Photos of tumors isolated from mice analyzed on day 20. n = 3 per group. **(C-E)** Cells isolated from tumor on day 20 were subjected to flow cytometry analysis. **(C, D)** The abundance of tumor-infiltrating OT-I T cells (CD8^+^CD45.1^+^CD45.2^+^) were analyzed. **(C)** Graph shows percentages of OT-I T cells. n = 6 (control-Crispr), n = 7 (Epas1-Crispr). **(D)** Graph shows absolute numbers of OT-I T cells per tumor volume (mm^3^). n = 7 per group. **(E)** Tim3 expression was analyzed. Graph shows MFI for Tim3 expression. n = 3 per group. Error bars indicate SD. Statistical analysis was performed using unpaired t-tests. *p <0.05, **p < 0.01. **(C-E)** Data are representative of 2 independent experiments. **(F)** On day 20 after tumor injection, OT-I T cells (CD8^+^CD45.1^+^CD45.2^+^) were FACS-sorted from tumors and subjected to bulk RNA-seq analysis. The volcano plot shows DEGs between OT-I T cells with Epas1-Crispr and control-Crispr (log2 fold change > 0.5 or < -0.5 and adjusted p < 0.05). Red and blue colored dots represent upregulated and downregulated genes in cells with Epas1-Crispr, respectively. n = 3 per group.

We next examined the impact of Epas1 deficiency on *in vivo* anti-tumor activity of CD8 T cells. We activated and expanded OT-I T cells electroporated with Epas1-Crispr or control-Crispr and adoptively transferred them to congenic C67BL/6 mice bearing B16-OVA tumors (**Figure 7A**). Suppression of tumor growth was less significant in mice transferred with OT-I T cells treated with Epas1-Crispr compared to controls (**Figures 7A, B**). Flow cytometry analysis revealed that the frequency of Epas1-Crispr-treated OT-I T cells in tumor-infiltrating lymphocytes was significantly lower than in controls (**Figures 7C, D**, **Supplementary Figure S5A**). Additionally, Epas1-Crispr treatment significantly increased expression of Tim3 in tumor-infiltrating OT-I T cells (**Figure 7E**). These results suggest that Epas1 promotes anti-tumor activity of CD8 T cells *in vivo*.

To understand how Epas1 promotes the anti-tumor response of CD8 T cells, we performed bulk RNA-seq analysis of tumor-infiltrating OT-I T cells treated with Epas1-Crispr. On day 12 after the adoptive transfer of OT-I T cells with Epas1-Crispr or control-Crispr into young recipient mice with B16-OVA tumors, we collected tumor-infiltrating lymphocytes and sorted OT-I T cells. Bulk RNA-seq analysis of these cells identified 32 differentially expressed genes (DEGs) between Epas1-Crispr and control-Crispr samples with a log2 fold change >|0.5| and adjusted p-value < 0.05 (**Figure 7F**). Genes downregulated by Epas1-Crispr included *G protein coupled-receptor 108* (*Gpr108*) and *coiled-coil domain containing 22* (*Ccdc22*), which are associated with the NF-κB pathway (40, 41); thiosulfate sulfurtransferase-like-domain-containing 2 (Tstd2), which promotes T cell infiltration of tumors (42); and Cd70, which facilitates T cell proliferation (43). In contrast, genes upregulated by Epas1-Crispr included polo-like kinase 4 (Plk4), which inhibits immune cell infiltration of tumors (44); and GRAM domain containing 4 (GRAMD4), which promotes apoptosis (45). Thus, Epas1 appears to control expression of several genes involved in infiltration of tumors, proliferation, and survival of CD8 T cells in anti-tumor responses.

**Figure 8:**
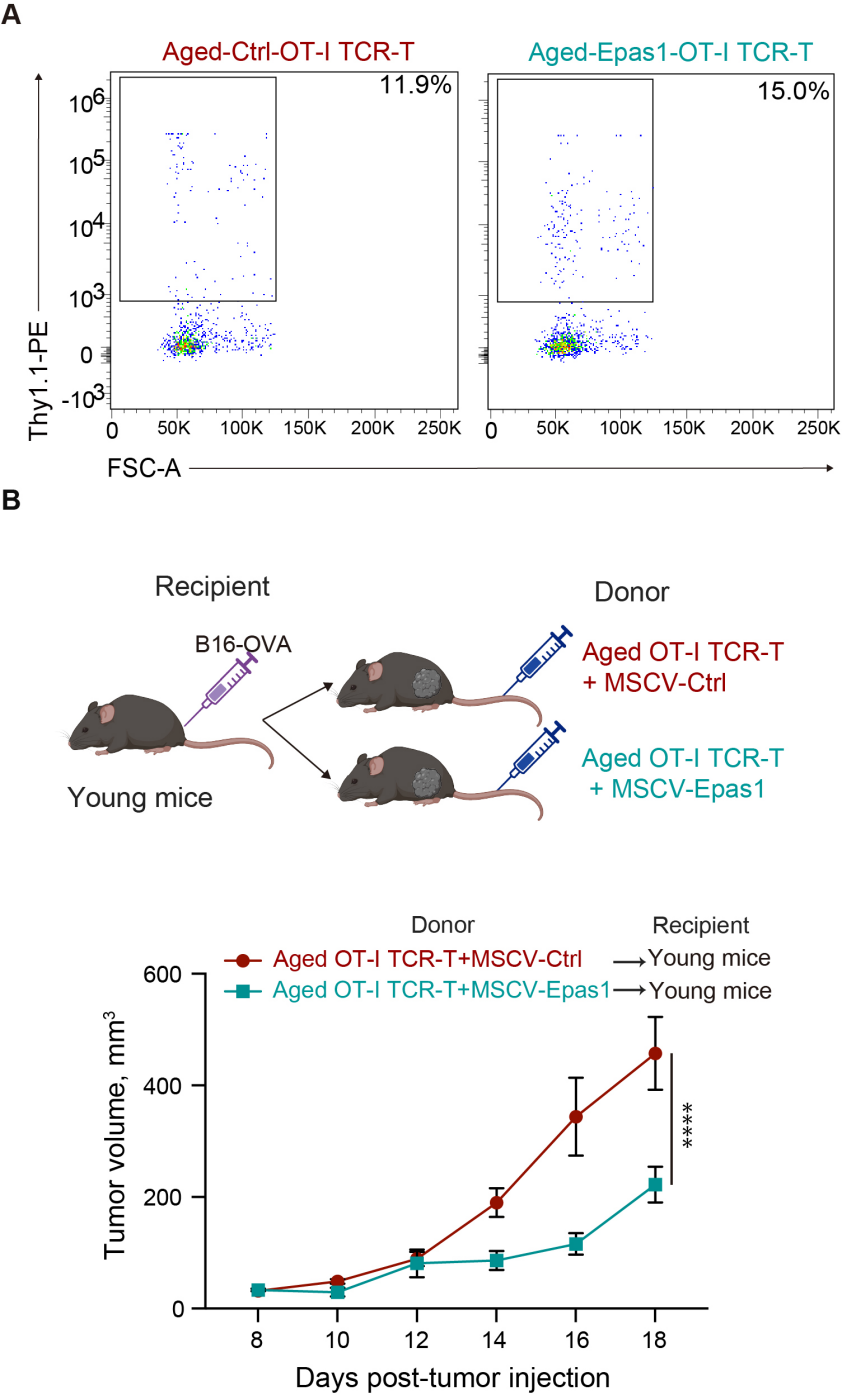
Retroviral expression of Epas1 enhances anti-tumor activity of CD8 TCR-T cells **(A, B)** CD8 T cells isolated from aged mice were transduced with a retroviral vector expressing Epas1 and Thy1.1 marker (MSCV-Epas1-Thy1.1) or control vector (empty MSCV-Thy1.1), followed by MIGR1-OT-I, generating aged OT-I TCR-T + MSCV-Epas1 cells and aged OT-I TCR-T + MSCV-control cells, respectively. Cells were cultured in the presence of IL-7 and IL-15 for 6 days. **(A)** Flow cytometry analysis of the frequency of cells transduced with MSCV-Epas1-Thy1.1 (Thy1.1^+^) in cells transduced with MIGR1-OT-I (GFP^+^). Representative plots are shown. **(B)** Cells including 10^6^ GFP^+^ cells were adoptively transferred into young mice that had been subcutaneously injected with 2 x 10^5^ of B16-OVA cells 8 days earlier (day 0). Left schematic diagrams show the experimental design (created with BioRender.com). Right graph shows results of tumor volume measurements. n=3 per group. Results are shown as mean tumor volume ± SEM. Statistical analysis was performed using two-way RM ANOVA with uncorrected Fisher’s LSD test. ****p<0.0001. **(A, B)** Data are representative of 2 independent experiments.

Finally, we tested whether expression of exogenous Epas1 enhances anti-tumor activity of aged CD8 TCR-T cells as reported in young CD8 T cells (39). We infected aged CD8 T cells with a retroviral vector expressing Epas1 and Thy1.1 marker, followed by another retroviral vector expressing OT-I TCR and GFP. Flow cytometry analysis confirmed the transduction efficiency of Epas1-expressing vector indicated by expression of Thy1.1 marker was about 15% of GFP^+^ cells transduced with OT-I TCR (**Figure 8A**). We adoptively transferred these cells into young mice bearing B16-OVA tumors. Notably, adoptive transfer of aged OT-I TCR-T cells with Epas1 transduction significantly inhibited tumor growth compared to that of control cells (**Figure 8B**). Additionally, mice transferred with aged TCR-T cells that were transduced with Epas1-expressing vectors showed weight changes comparable to those of the control group (**Supplementary Figure S6**). Taken together, these results suggest that decreased expression of Epas1 impairs anti-tumor activity of CD8 TCR-T cells in ACT, which can be improved by expression of exogenous Epas1.

## Discussion

This study demonstrated that aging significantly impairs anti-tumor responses of antigen-specific CD8 T cells in ACT using a mouse model of melanoma. We found that aged CD8 T cells retrovirally transduced with TCRs specific to tumor antigens can proliferate and maintain anti-tumor cytolytic capacity *in vitro*, but that their anti-tumor activity *in vivo* is diminished. Age-related CD8 T-cell-intrinsic changes are sufficient to impair their anti-tumor responses after adoptive transfer into young recipient mice. Moreover, we identified *Epas1* as a representative gene downregulated in aged TCR-T cells. Importantly, Epas1 deficiency restricts the capacity of young CD8 T cells to accumulate as Tpex-like cells in tumors, so as to control tumor growth. In contrast, retroviral expression of Epas1 promotes anti-tumor activity of aged CD8 T cells in ACT. These results imply that decreased expression of Epas1 is associated with diminished anti-tumor activity of aged CD8 TCR-T cells.

We found that both aging and Epas1 deficiency impair accumulation of TCR-T cells in tumors, which likely causes the decreased anti-tumor activity of these cells. Our scRNA-seq data for intra-tumoral TCR-T cells revealed the age-related decrease in the frequency of Mki67-expressing Tpex-like cells (cluster 0), which can persist in tumors and sustain effective anti-tumor CD8 T cell responses more effectively than Tex cells (46, 47). Meanwhile, we observed a slight increase in the proportions of Tex-like cells (clusters 3 and 4), cells differentiating to Tex (clusters 1 and 2), and cells lacking effector molecule expression (cluster5). Furthermore, we observed that intra-tumoral Epas1-deficient TCR-T cells exhibit increased expression of Tim3, which suggesting a phenotypic shift from Tpex-like cells to Tex cells, along with reduced expression of regulators of cell proliferation, such as Myc. These results imply that impaired intra-tumoral accumulation of aged TCR-T cells may be due to their defects in Epas1-mediated induction or maintenance of Tpex-like cells with high proliferative capacity.

Our results also suggest that defective anti-tumor activity of aged TCR-T cells may not be solely on decreased Epas1 expression. For example, our *in vitro* analysis revealed that aging, but not Epas1 deficiency, impairs CD8 TCR-T cell capability to exhibit a central memory phenotype, which is associated with high anti-tumor activity (31). Additionally, we observed that aging, but not Epas1 deficiency, significantly increases GzmB expression in TCR-T cells, without affecting their cytotoxic activity *in vitro*. Since GzmB primarily exerts cytotoxic functions in a perforin-dependent manner (48), age-related increase in GzmB expression alone is not likely sufficient to enhance cytotoxic activity of aged CD8 TCR-T cells.

Hif transcription factors, Epas1 and Hif1α, appear to have shared and distinct functions in CD8 T cell responses. Hif1α, but not Epas1, is necessary for *in vitro* CD8 T cell responses, including TCR-induced metabolic reprogramming to glycolysis and the hypoxia-induced increase in expression of effector molecules, such as IFN-γ and GzmB (49). Moreover, Hif1α is indispensable for anti-tumor responses of both endogenous CD8 T cells and adoptively transferred tumor-specific CD8 T cells (50). In contrast, Epas1 is required for anti-tumor responses of adoptively transferred tumor-specific CD8 T cells, as we demonstrated, but not for anti-tumor responses of endogenous CD8 T cells (50). Our observation of altered frequencies of Tim3-expressing cells in intra-tumoral *Epas1*-deficient CD8 T cells suggests that Epas1 is involved in control of the balance between Tpex and Tex cells. In contrast, it remains unknown whether loss of Hif1α causes similar effects in intra-tumoral CD8 T cells. Epas1 might also promote infiltration of tumor-specific T cells into tumors, like Hif1a (50), since Epas1 controls expression of genes involved in tumor infiltration of immune cells, such as *Plk4* and *Tstd2*.

How *Epas1* mRNA expression is decreased by aging remains unknown. Age-related decrease in *Epas1* expression was also observed in gingival tissues of monkeys (51) and in the lungs of sheep (52), but the underlying mechanism was not studied. Although hypoxia-mediated protein stabilization is the major mechanism to regulate expression of Epas1 and Hif1α (53), transcriptional regulatory mechanisms also help to control their expression (54). Since aging decreases mRNA expression of *Epas1*, but not *Hif1a*, aging should lead to defects in transcriptional regulatory mechanisms specific to *Epas1*. As TCR signaling promotes expression of both *Epas1* and *Hif1* (50, 55), the age-related decrease in TCR signaling (56) is not likely the main cause of this specific reduction of *Epas1* expression. *Epas1* transcription is promoted by distinct pathways mediated by IL-4 (57), mTORC2 (58), and E2F transcription factor (59), and it is inhibited by an epigenetic mechanism mediated by histone deacetylase (HDAC) (60) and DNA methyltransferase 3 alpha (DNMT3a) (61). In our GSEA of scRNA-seq data, genes related to the E2F pathway were significantly downregulated in aged CD8 T cells. Thus, it is possible that age-related downregulation of E2F is responsible for decreased expression of Epas1 in aged CD8 T cells.

Using an ACT model against melanoma in female mice, this study revealed the impact of aging and modulation of Epas1 expression on anti-tumor responses of CD8 T cells. Despite considerable variations in tumor growth kinetics observed in the same group of samples, likely due to technical variations in the preparation of TCR-T cells, we detected statistically significant differences indicating the effects of aging and knockout or overexpression of Epas1 in CD8 T cells on the tumor growth. However, since we used only female mice for the *in vivo* experiments, future studies need to determine the relevance of our findings in other experimental settings. It is important to assess whether age-related changes and Epas1 functions in CD8 T cells in adoptive cell therapy are conserved across both sexes and in diverse organisms, including humans. Furthermore, verifying Epas1 decrease in CD8 T cells in cancer patients is helpful to explore the therapeutic potential of Epas1 overexpression in ACT with aged CD8 T cells. It is also interesting to investigate the effects of aging and Epas1 deficiency on anti-tumor responses of CD4 T cells.

In summary, our findings highlight negative impacts of aging on ACT for solid tumors using tumor-specific CD8 T cells. Aged CD8 T cells have reduced expression of Epas1, which promotes anti-tumor CD8 T cell responses by enhancing intra-tumoral accumulation of Tpex cells. Thus, Epas1 may be a biomarker to predict the age-related decline in anti-tumor efficacy of ACT using tumor-specific CD8 T cells. It may also serve as a potential therapeutic target to improve such therapy in elderly patients.

## Data Availability

The datasets presented in this study can be found in online repositories. The names of the repository/repositories and accession number(s) can be found below: https://www.ddbj.nig.ac.jp/, PRJDB18653.
