# Supplementary material for: Aging impairs CD8 T cell responses in adoptive T-cell therapy against solid tumors
Source: Front Immunol. 2025 Jan 24;16:1484303. doi: 10.3389/fimmu.2025.1484303 (PMC11803149; doi:10.3389/fimmu.2025.1484303)
Supplement: Supplementary file 1 [file DataSheet1.pdf]

Supplementary Fig. 1.

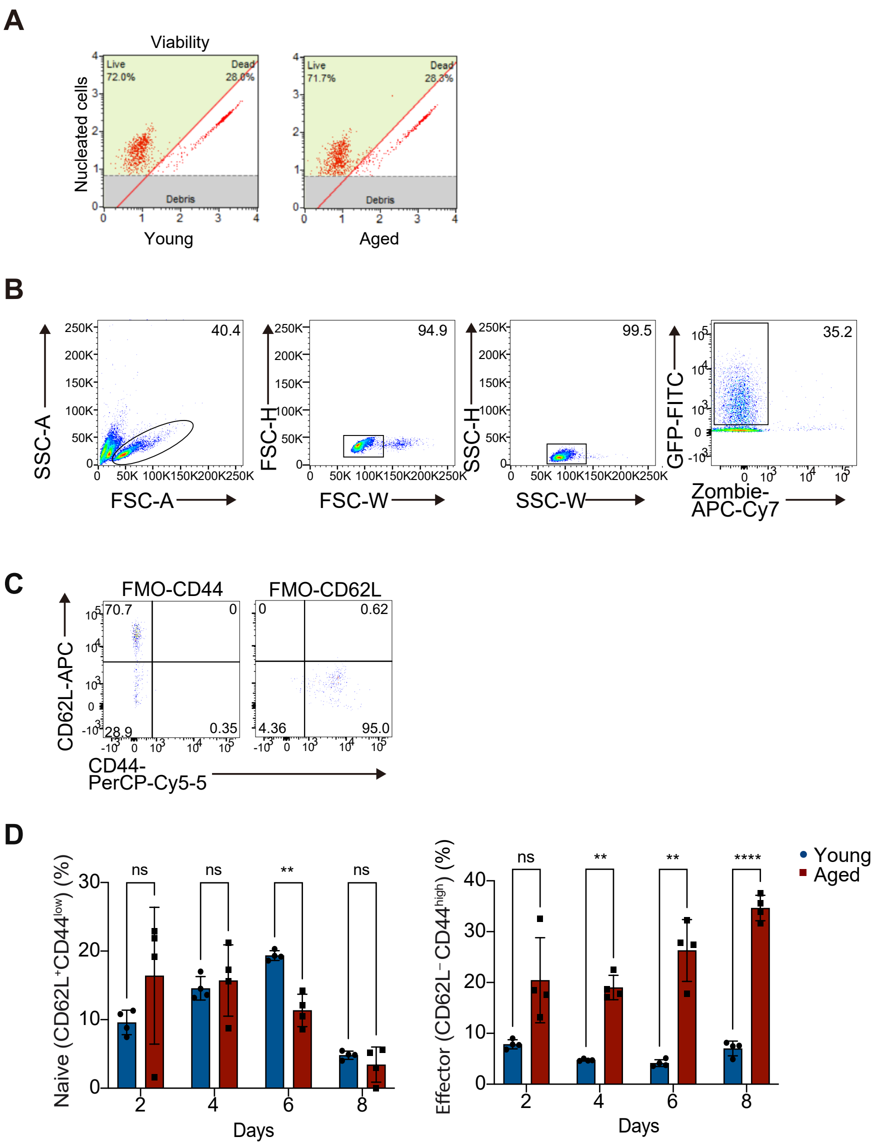

Supplementary Fig. 1. Analysis of CD8 TCR-T cells in vitro (related to Fig. 1).

(A) Muse plots showing percentages of living cells detected in the green marked area. Related to Fig. 1A, B. **(B)** Gating strategy for analysis of GFP^{+} OT-I TCR-T cells. **(C)** Representative plots showing fluorescence minus one (FMO) for analysis of CD44 and CD62L expression. **(D)** Graphs show percentages of cells expressing $CD62L^{-}$ $CD44^{hi}$ effector cells and $CD62L^{+}$ $CD44^{lo}$ naive cells. Statistical analysis was performed using RM two-way ANOVA with an uncorrected Fisher's LSD test. **(C, D)** Related to Fig. 1D.

Supplementary Fig. 2.

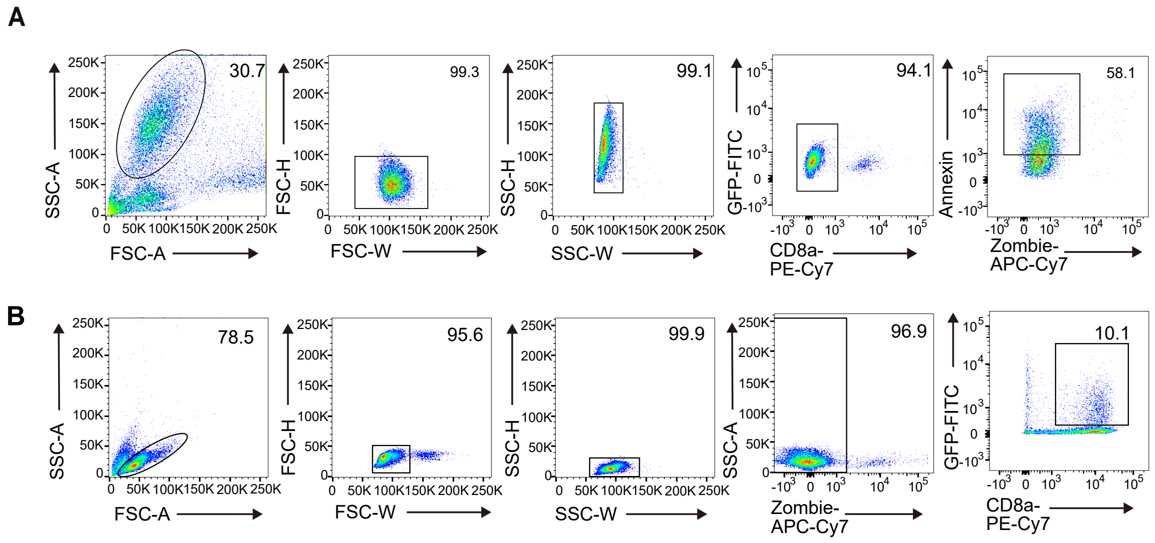

Supplementary Fig. 2. Flow cytometry analysis of cells in co-culture experiments (related to Fig. 2). (A, B) Gating strategy for B16-OVA cells (A) and OT-I TCR-T cells (B).

Supplementary Fig. 3.

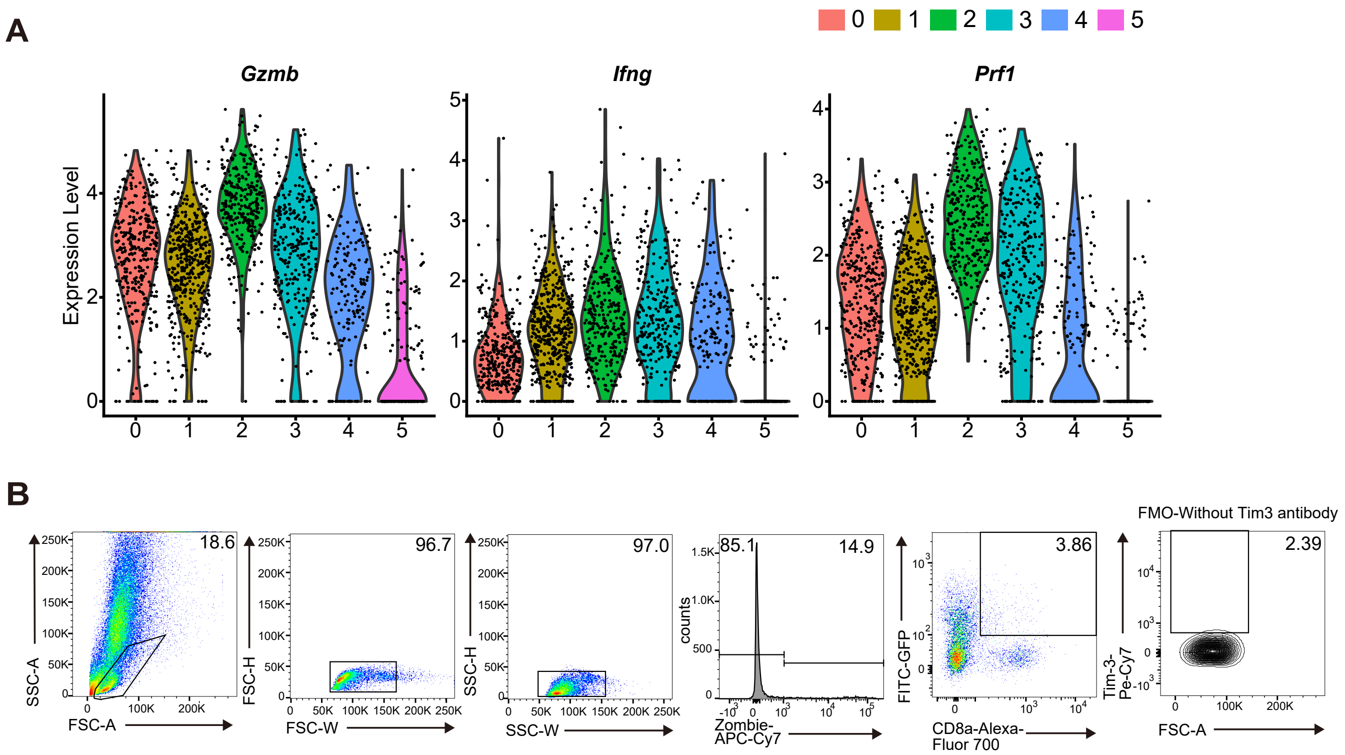

Supplementary Fig. 3. Analysis of OT-I TCR T cells isolated from tumor tissues (related to Fig. 4). (A) Violin plots show expression of cytokines (Gzmb, Ifng, and Prf1) in clusters shown in Fig. 4. **(B)** Gating strategy for $CD8^{+}GFP^{+}$ OT-I TCR-T cells in tumor tissues.

Supplementary Fig. 4.

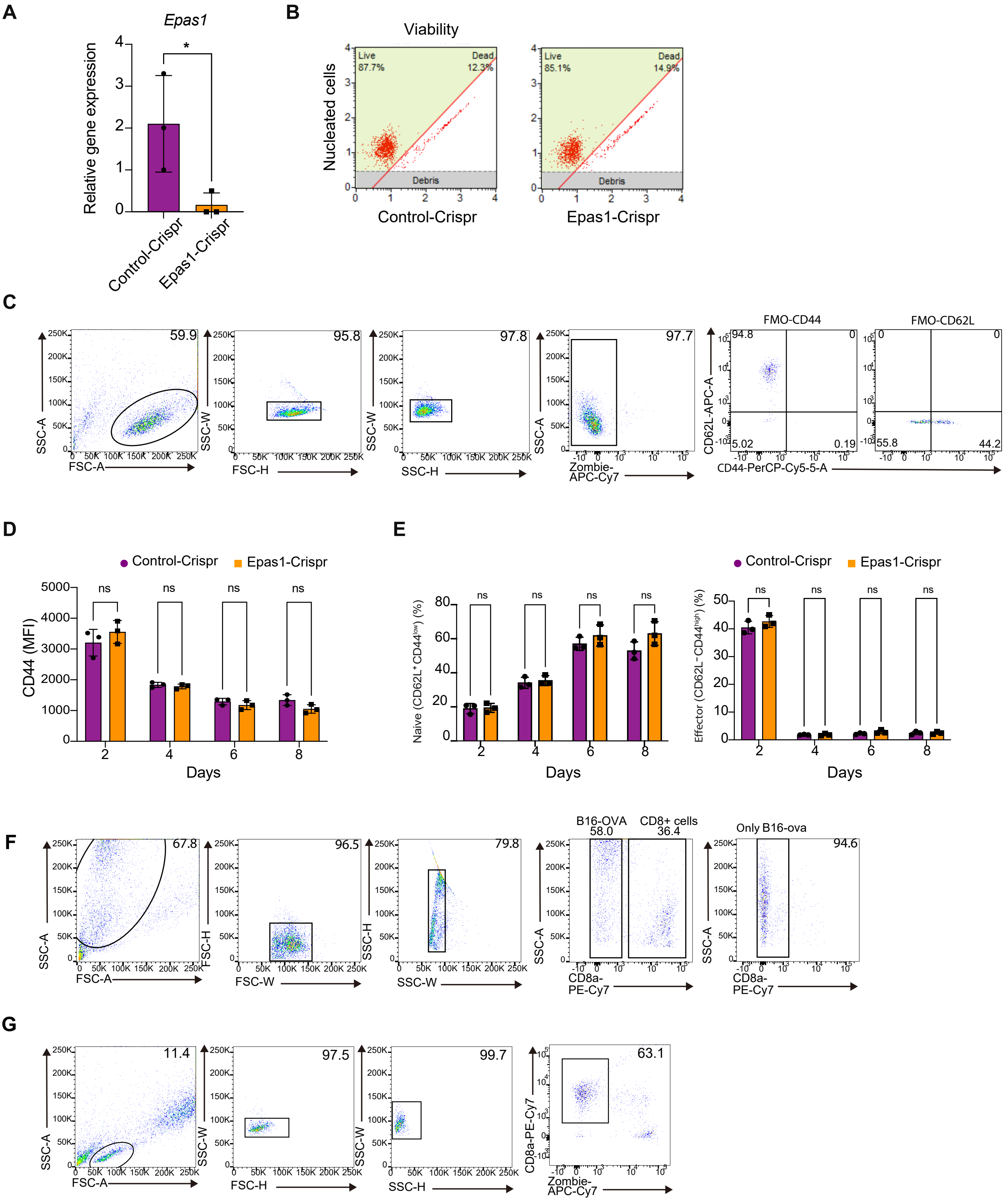

Supplementary Fig. 4. Analysis of OT-I cells treated with Epas1-Crispr (related to Fig. 6). (A) qPCR analysis of Epas1 mRNA in young OT-I T cells 3 days after treatment with Epas1-Crispr. n = 3 per group. Statistical analysis was performed with paired t-tests (* $p < 0.05$). Data are representative of 2 independent experiments. **(B)** Muse plots showing percentages of living cells detected in the green marked area. Related to Fig. 6A, B. **(C)** Gating strategy for analysis of CD44 and CD62L expression. **(D)** Graph shows MFI of CD44 expression (related to Fig. 6C). **(E)** Graphs show percentages of cells expressing $CD62L^{-}$ $CD44^{hi}$ effector cells and $CD62L^{+}$ $CD44^{lo}$ naive cells (related to Fig. 6C). Statistical analysis was performed using RM two-way ANOVA with an uncorrected Fisher's LSD test. **(F)** Gating strategy for B16-OVA cells or $CD8^{+}$ cells. **(G)** Gating strategy for $CD8^{+}$ cells.

Supplementary Fig. 5.

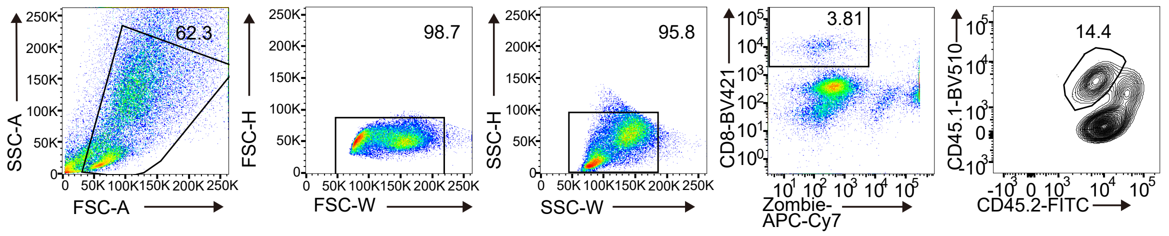

Supplementary Fig. 5. Analysis of OT-I cells with Epas1-Crispr in vivo (related to Fig. 7).

Gating strategy for analysis of OT-I cells (CD8⁺ CD45.1⁺ CD45.2⁺).

Supplementary Fig. 6.

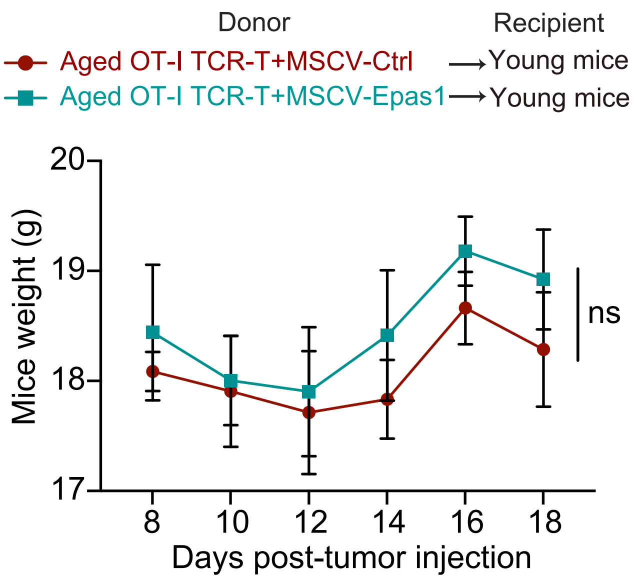

Supplementary Fig. 6. Analysis of mice transplanted with B16-OVA cells followed by adoptive transfer of Epas1-overexpressing OT-I T cells (related to Fig. 8). The body weights of mice were measured. n = 3 per group Results are shown as mean body weights ± SEM. Statistical analysis was performed using two-way RM ANOVA with uncorrected Fisher's LSD test. ns: not significant.
